# Molecular cytological analysis of alien introgressions in common wheat lines created by crossing of Triticum aestivum with T. dicoccoides and T. dicoccum

**DOI:** 10.18699/VJGB-23-67

**Published:** 2023-10

**Authors:** О.A. Orlovskaya, I.N. Leonova, L.A. Solovey, N.I. Dubovets

**Affiliations:** Institute of Genetics and Cytology of the National Academy of Sciences of Belarus, Minsk, Belarus; Institute of Cytology and Genetics of the Siberian Branch of the Russian Academy of Sciences, Novosibirsk, Russia Department of Genetics and Selection, Novosibirsk State Agricultural University, Novosibirsk, Russia; Institute of Genetics and Cytology of the National Academy of Sciences of Belarus, Minsk, Belarus; Institute of Genetics and Cytology of the National Academy of Sciences of Belarus, Minsk, Belarus

**Keywords:** common wheat, Triticum aestivum, T. dicoccoides, T. dicoccum, introgression lines, C-banding, SSR analysis, SNP analysis, microsporogenesis, cytological stability, мягкая пшеница, Triticum aestivum, T. dicoccoides, T. dicoccum, интрогрессивные линии, C-бэндинг, SSR-анализ, SNP-анализ, микроспорогенез, цитологическая стабильность

## Abstract

Wild and domesticated emmer (ВВАА, 2n = 28) are of significant interest for expanding the genetic diversity of common wheat as sources of a high protein and microelement grain content, resistance to many biotic and abiotic factors. Particular interest in these species is also determined by their close relationship with Triticum aestivum L., which facilitates interspecific hybridization. The objective of this work was to analyze the nature of alien introgressions in hybrid lines from crossing common wheat varieties with T. dicoccoides and T. dicoccum, and to assess the effect of their genome fragments on the cytological stability of introgression lines. A C-banding technique and genotyping with SNP and SSR markers were used to determine localization and length of introgression fragments. Assessment of cytological stability was carried out on the basis of chromosome behavior in microsporogenesis. A molecular cytogenetic analysis of introgression wheat lines indicated that the inclusion of the genetic material of wild and domesticated emmer was carried out mainly in the form of whole arms or large fragments in the chromosomes of the B genome and less extended inserts in the A genome. At the same time, the highest frequency of introgressions of the emmer genome was observed in chromosomes 1A, 1B, 2B, and 3B. The analysis of the final stage of meiosis showed a high level of cytological stability in the vast majority of introgression wheat lines (meiotic index was 83.0–99.0 %), which ensures the formation of functional gametes in an amount sufficient for successful reproduction. These lines are of interest for the selection of promising material with agronomically valuable traits and their subsequent inclusion in the breeding process.

## Abstract (ru)

Дикая и культурная полбы (ВВАА, 2n = 28) представляют значительный интерес для расширения генетического разнообразия мягкой пшеницы как источники генетических факторов, определяющих высокое содержание белка и микроэлементов в зерне и устойчивость ко многим биотическим и абиотическим факторам. Особое внимание к этим видам обусловлено их близким родством с Triticum aestivum, что облегчает межвидовую гибридизацию. Целью настоящей работы были анализ характера чужеродных интрогрессий у гибридных линий, полученных от скрещивания сортов мягкой пшеницы с T. dicoccoides и T. dicoccum, и оценка их влияния на цитологическую стабильность. Для установления локализации и протяженности фрагментов интрогрессии использовали С-бэндинг и генотипирование маркерами SNP и SSR. Оценку цитологической стабильности проводили на основе изучения поведения хромосом в микроспорогенезе. Молекулярно-цитогенетический анализ интрогрессивных линий пшеницы свидетельствует о том, что включение генетического материала дикой и культурной полб осуществляется преимущественно в виде целых плеч или крупных фрагментов в хромосомах генома В и менее протяженных вставок в геноме А. При этом наибольшая частота интрогрессий фрагментов генома полб наблюдалась в хромосомах 1А, 1В, 2В, 3В. Изучение заключительной стадии мейоза показало вы- сокий уровень цитологической стабильности у большинства интрогрессивных линий пшеницы (мейотический индекс составил 83.0–99.0 %), что обеспечивает формирование у них функциональных гамет в количестве, достаточном для успешной репродукции. Данные линии представляют интерес для отбора перспективного материала с хозяйственно ценными признаками с последующим включением их в селекционный процесс.

## Introduction

Common wheat Triticum aestivum L. (BBAADD, 2n = 42) is one of the most important cereal crops and a major source of calories for most of the world’s population. In addition to food purposes, wheat is used in the pulp and paper and chemical industries, for the production of ethanol, and flour milling waste and feed grains are used as livestock feed. Breeding bread wheat for high-yielding varieties has significantly reduced the level of genetic diversity compared to wild relatives (Xie, Nevo, 2008; Nevo, Chen, 2010; Budak et al., 2013). At present, other species of the genus Triticum are increasingly used to expand the bread wheat gene pool (Jaradat, 2013; Liu et al., 2019; Orlovskaya et al., 2020).

Wild tetraploid wheat, or wild emmer wheat T. dicoccoides Schwein f. (BBAA, 2n = 28) appeared as a result of spontaneous hybridization between diploid species: T. urartu Thum. (AA, 2n = 14) and unknown close relative of Aegilops speltoides Tausch. (SS, 2n = 14) (Dvorak et al., 1993; Peng et al., 2011). It is assumed that wild emmer participated in the formation of domesticated emmer T. dicoccum (Schrank.) Schuebl (BBAA, 2n = 28). The formation of common wheat occurred as a result of natural hybridization of a tetraploid species from the genus Triticum (BBAA) and a diploid species Ae. tauschii Coss., a D genome donor (Petersen et al., 2006).

Wild and domesticated emmer are of significant interest in expanding the genetic diversity of common wheat. Many accessions of T. dicoccoides are known to be adapted to unfavorable environments (Peleg et al., 2005; Nevo, Chen, 2010), characterized by a high protein and microelements content (Cakmak et al., 2004; Uauy et al., 2006; Wang Z. et al., 2018). More than 20 genes and QTLs have been identified in the genomes of T. dicoccum and T. dicoccoides for resistance to powdery mildew, leaf rust, yellow rust, and Fusarium (Peng et al., 2000; Xie, Nevo, 2008). Interest in wild and domesticated emmer is also due to phylogenetic relationship with common wheat. However, despite the closeness of emmer genomes to common wheat genomes A and B, the transfer of alien chromatin into cultivars may be accompanied by introgression of genetic material that negatively affects agronomically important traits.

Cytological and molecular methods are effective tools for chromosomal identification of foreign chromatin in the common wheat genome. One of them is differential staining of mitotic chromosomes (C-banding), which makes it possible, based on a comparison of C-banding patterns in hybrids and initial parental forms, to reveal structural transformations of the karyotype, indicating the introgression of foreign chromatin. However, many cereal genomes contain an insignificant amount of heterochromatin, and closely related species often have a similar C-banding pattern, which limits the application of this method (Surzhikov et al., 2007; Dedkova et al., 2009).

Molecular markers are effective for detecting of structural changes in low-heterochromatin genomes and identifying of short introgressed fragments, among which SSR and SNP markers are most widely used (Zhou et al., 2013; Jorgensen et al., 2017). At present molecular genetic maps of the chromosomes of hexaploid wheat and wild emmer have been constructed based on SSR and SNP markers specific for the A, B, and D genomes of T. aestivum (Röder et al., 1998; Pestsova et al., 2000; Wang S. et al., 2014; Maccaferri et al., 2015). The use of molecular markers increases the efficiency of foreign introgression monitoring.

Previously, we studied the nature of foreign substitutions and translocations and the process of stabilization of hybrid lines obtained by crossing common wheat varieties with T. kiharae (AtAtGGDD, 2n = 42) (Orlovskaya et al., 2020). It has been shown that the introgression of the genetic material of T. kiharae occurs as whole chromosomes or large fragments (centric and terminal translocations). The objective of this work was to analyze the nature of alien introgressions in hybrid lines obtained from hybridization of common wheat varieties with T. dicoccoides and T. dicoccum, and to assess their effect on the cytological stability of introgression lines.

## Materials and methods

We used nine F10 lines obtained at the Institute of Genetics and Cytology of the National Academy of Sciences of Belarus, from crossing common wheat varieties T. aestivum (Rassvet, Festivalnaya, and Pitic S62) with emmer accessions from the N.I. Vavilov All-Russian Institute of Plant Genetic Resources (VIR) collection T. dicoccoides k-5199 and T. dicoccoides (the origin of this accession is unknown), and T. dicoccum k-45926. Line 29 (Rassvet × T. dicoccoides k-5199); lines 11-1, 13-3, 15-7-2, and 16-5 (T. dicoccoides × Festivalnaya); lines 206-2-2 and 213-1-2 (Pitic S62 × T. dicoccum k-45926) and lines 1-3 and 2-7 (Festivalnaya × T. dicoccum k-45926) were created by self-pollination of F1 hybrids and subsequent generations and selected for molecular cytogenetic studies based on the assessment of the inheritance of morphological and productivity traits in F1–F9 generations.

The preparation of cytological plates and the procedure of C-banding were performed according to the method of E.D. Badaeva et al. (1994). Identification of individual chromosomes of A-, B- and D-genomes was carried out in accordance with the generalized ideogramme of differentially stained chromosomes developed by E.D. Badaeva et al. (1990). Stained slides were analyzed using Amplival microscope (Carl Zeiss, Jena, Germany). Selected metaphase plates were photographed using the Leica DC 300 digital video camera. Processing of the obtained images was carried out using graphics editor Adobe Photoshop 2017.

Genomic DNA was isolated from the seedlings of 5–7-days old as described in E.S. Skolotneva et al. (2017). Genotyping with SNP markers was performed using Illumina Infinium 20K chip technology (TraitGenetics, Germany, http://www. traitgenetics.com). SSR markers (WMC, GWM, and GDM) were used to clarify the chromosomal location and the length of introgression fragments, using two or more markers per chromosome arm. Polymerase chain reaction (PCR) protocols for SSR markers are described in M.S. Röder et al. (1998). Separation of PCR fragments was performed on an ABI PRISM 3100 automatic sequencer (Applied Biosystems, USA). The fragment size was calculated using the ABI GeneScan software (version 2.1). Putative chromosomal localization was determined based on wheat chromosome consensus maps constructed using SSR and SNP markers (Somers et al., 2004; Wang S. et al., 2014).

Microsporogenesis was studied on temporary squashed preparations. Spikes were cut before leaving the leaf sheath and fixed in the ethanol-acetic mixture (3:1). A day after fixation, the material was transferred to 70 % ethyl alcohol, where it was stored before analysis at t = +2–4 °С. Acetoorcein (2 %) was used as a dye. For each cross combination and initial forms, 30 plates of metaphase I and 50–100 microsporocytes of the following stages of meiosis (anaphase I and II, metaphase II, tetrads) were analyzed. The slides were analyzed on the microscope Amplival (Carl Zeiss) with Apochromate lens 100x aperture 1.32 MI.

Statistical data analysis was carried out using STATISTICA v. 10 (http://statsoft.ru/) and MS Excel 2010.

## Results

C-banding

In this study, karyotyping of five hybrid lines obtained with the involvement of common wheat cultivars and two accession of T. dicoccoides was carried out. Of these, two lines (11-1 and 13-3) were tetraploid (2n = 4x = 28), while others were stabilized at the hexaploid ploidy level (2n = 6x = 42).

Both T. dicoccoides accessions had an almost identical C-banding pattern, while differences between varieties were observed in the degree of expression and the presence/absence of a number of telomeric and intercalary blocks of heterochromatin, which ensured the individuality of their karyotypes. The vast majority of these differences were found in the heterochromatin-rich genome B. As for the A-genome, polymorphism between parental forms was noted only in two chromosomes – 4A and 6A, and only in the T. dicoccoides × Festivalnaya cross combination: chromosome 4A of T. dicoccoides has a large telomeric block of heterochromatin in the long arm, while Festivalnaya has a more pronounced subtelomeric block. The 6A chromosome of T. dicoccoides is also distinguished by the presence of a bright intercalary block in the proximal region of the long arm. Comparison of the obtained patterns of differential chromosomes staining in hybrid lines of wheat and the corresponding parental forms made it possible to identify the presence of introgressions of the genetic material of T. dicoccoides in all five lines (Table 1).

**Table 1.**
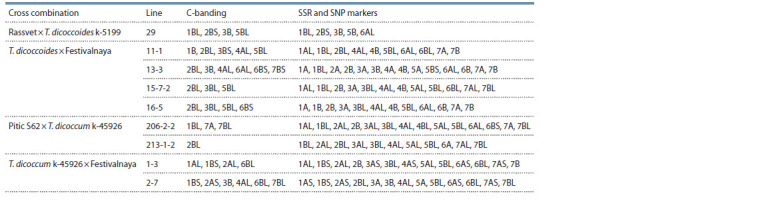
Chromosomal localization of emmer genetic material in introgression wheat lines according to C-banding and genotyping data using SSR and SNP markers

**Fig. 1.**
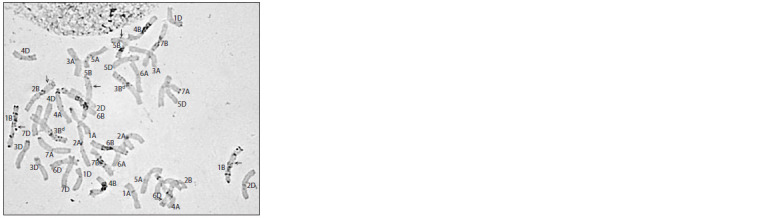
Karyotype of introgression wheat line 29 Rassvet × T. dicoccoides k-5199. Here and in Fig. 2: locations of introgressed fragments of wild emmer are indicated by arrows.

In line 29, the distribution of heterochromatin blocks characteristic of wild emmer was noted in the proximal region of the long arm of chromosome 1B and in the distal regions of the short arm of chromosome 2B and the long arm of 5B (Fig. 1).

It is not possible to determine the size of the T. dicoccoides genome fragments included in the cv. Rassvet genome due to the identity of the C-banding patterns of the parental forms in the adjacent regions of the chromosomes. In this regard, in Fig. 1 and in all subsequent Figures depicting the karyotypes of the studied lines, arrows mark only the localization sites of alien fragments. Changes in the C-banding pattern were also detected in chromosome 3B, both in the long and short arms, which gave us reason to assume that in this case, the whole chromosome of T. dicoccoides was introgressed. As for chromosome 4B, in all parental forms this chromosome had an identical pattern of staining, which did not allow us to conclude the possible fact of the genetic material exchange between these homoeologs of wheat and T. dicoccoides in any of the hybrid lines.

**Fig. 2.**
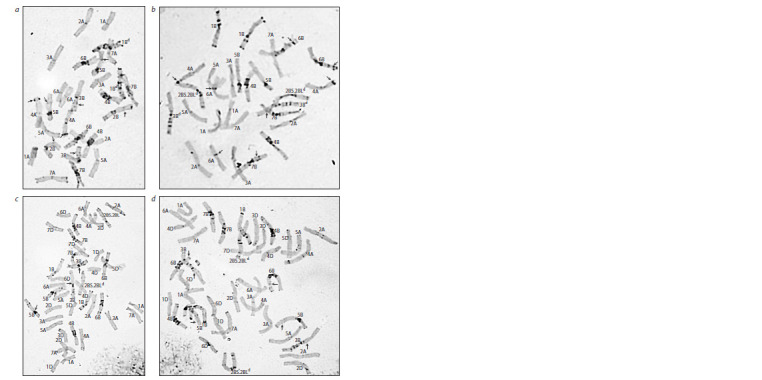
Karyotypes of introgression wheat lines of T. dicoccoides × Festivalnaya cross combination. Lines: a – 11-1; b – 13-3; c – 15-7-2; d – 16-5. Intact arms of T. dicoccoides are indicated with a superscript “d”.

In the karyotype of line 11-1, the C-banding pattern typical for T. dicoccoides was observed in the distal region of the long arm of chromosome 4A, as well as in the central regions of the short arm of 3B and long arms of chromosomes 2B and 5B (Fig. 2, a). Taking into account that 4A homoeolog of wild emmer and Festivalnaya differ only in the C-banding pattern of the distal region of the long arm, it is not possible to infer unequivocally whether the whole emmer chromosome, or the long arm, or only its distal part was introgressed. At the same time, chromosome 1B, judging by the distribution of heterochromatin blocks in both arms, most likely belongs entirely to T. dicoccoides. In other cases, the insertion of emmer chromatin fragments is more likely.

The karyotype of line 13-3 (see Fig. 2, b) demonstrated changes in the C-banding pattern in the distal region of the long arm of chromosome 4A similar to changes in line 11-1. In addition, chromosome 6A has a bright intercalary block typical to T. dicoccoides in the proximal region of the long arm. As for the chromosomes of the genome B, introgressions of T. dicoccoides were identified in 2BL (presumably the whole arm belongs to wild emmer), and in the distal 6BS and proximal 7BS regions. Chromosome 3B, according to the C- banding pattern, belongs entirely to wild emmer. Of particular note is the fact that this line contains a heteromorphic pair of chromosomes 6B, in which only one of the homoeologs is characterized by a change in the C-banding pattern in the distal region of the short arm. At the same time, the size of the introduced emmer fragment is unclear, since, as in the case of chromosome 4A, polymorphism in the distribution of heterochromatin blocks in 6B homologues was noted only in the above region.

For line 15-7-2, introgression of the T. dicoccoides genetic material was found in the long arm of chromosome 2B, as well as in the proximal regions of the long arms of chromosomes 3B and 5B (see Fig. 2, c).

A similar set of recombinant chromosomes (2B, 3B, and 5B) was found in line 16-5 with the only difference that chromosome 5B contained a fragment of emmer chromatin not from the proximal, but from distal region of the long arm. In addition, a fragment of T. dicoccoides chromatin was identified in the distal region of the short arm of chromosome 6B (see Fig. 2, d ).

It should be noted that introgression of the genetic material of T. dicoccoides into the genome of common wheat occurred in accordance with the homoeology of chromosomes. In the course of stabilization of karyotypes in each homoeologous group, identical variants of resulting recombinant chromosomes were selected, and as a result, all introduced fragments of the emmer genome are present in disomic state (the only exception is 6B chromosome in line 13-3). No chromatin exchanges between non-homoeologous chromosomes were found in the studied material

The study of the nature of introgressions of domesticated emmer T. dicoccum k-45926 into the genome of common wheat was carried out using the material of four hybrid lines developed with the involvement of wheat varieties Festivalnaya and Pitic S62. Analysis of the karyotype of the emmer wheat demonstrated the presence of the T7AL-5BS.5BL translocation resulting from the transfer of a fragment of the long arm of chromosome 7A to the distal region of the short arm of chromosome 5B. The other structural transformations were found in chromosome 7A with a deletion of the distal part of the long arm. According to the literature data, both types of aberrant chromosomes are widespread among T. dicoccum accessions growing in the Mediterranean and Western Europe (Dedkova et al., 2007). Similar structural rearrangements of chromosomes were also noted in some T. dicoccoides genotypes (Badaeva et al., 2007).

**Fig. 3.**
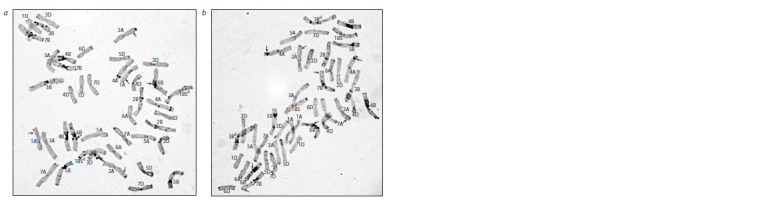
Karyotypes of introgression wheat lines of T. dicoccum k-45926 × Festivalnaya combination: a – line 1-3; b – line 2-7. Here and in Fig. 4: introduced chromosomes and whole arms of T. dicoccum k-45926 are marked with a superscript “d”. Arrows indicate location of introgression fragments of domesticated emmer

When comparing the obtained patterns of differential staining of chromosomes in hybrid wheat lines and corresponding parental forms, the presence of introgressions of the T. dicoccum genetic material was established in all the lines under study (see Table 1). In the karyotype of line 1-3 from the T. dicoccum × Festivalnaya cross combination, C-banding pattern typical for T. dicoccum was observed in the distal regions of long arms of chromosomes 1A, 2A, and 6B. The size of introgressed fragments was approximately equal to half of the arm. For chromosome 1B, the insertion of a short arm of domesticated emmer was identified (Fig. 3, a).

The second line from the same cross combination is characterized by a larger amount of introgressed emmer chromatin: in the karyotypes of all analyzed plants, emmer chromatin was found in chromosome 3B, in chromosome 1B, near telomeres of 6B and 7B chromosomes, in the distal region of chromosome 2A, and also the distal region of the long arm of chromosome 4A (see Fig. 3, b).

In the karyotype of line 206-2-2 from the cross combination Pitic S62 × T. dicoccum k-45926, chromosome 7A, characteristic of the original emmer accession, was present with a deletion of the distal fragment of the long arm. The similarity of C-banding patterns in the short arms of this chromosome in the parental wheat cultivar and emmer does not allow us to make an unambiguous conclusion whether this chromosome is recombinant or completely belongs to emmer. We are more inclined to the second option. In addition, the C- banding pattern typical of T. dicoccum k-45926 was noted in the distal regions of chromosomes 1BL and 7BL (Fig. 4, a). The smallest amount of T. dicoccum k-45926 genetic material (long arm of chromosome 2B) introduced into the wheat genome was found in line 213-1-2 (see Fig. 4, b). As in case of the introgression of the wild emmer genetic material, all introduced fragments of the domesticated emmer genome are present in the karyotypes of hybrid lines in the disomic state.

## Molecular analysis

The number of SNP markers mapped on different chromosomes of A-, B- or D-genomes varied significantly with the smallest number noted for chromosomes of the 4th homoeological group (Supplementary Material 1)1. More than 50 % of SNP markers of A and B genomes revealed polymorphism between T. dicoccum, T. dicoccoides and parental wheat varieties. A high amplification level of SNP markers of the D genome was also noted in T. dicoccum and T. dicoccoides. However, it is currently not possible to establish their chromosomal localization in the genome of wild and domesticated emmer.

Supplementary Materials are available in the online version of the paper: https://vavilov.elpub.ru/jour/manager/files/Suppl_Orlovskaya_Engl_27_6.pdf


Despite the high coverage of chromosomes with SNP markers, in the distal regions of some chromosomes (1AS, 1AL, 2BS, 3BL, 4AS, 4BL, 6AL, and 7AS) the number of polymorphic markers was insufficient to determine the completeness of substitution and fragment lengths. SSR markers were additionally used to correct the length of introgressed fragments. Analysis of the polymorphism of SSR markers indicates that almost all of the markers used are polymorphic, while the absence of amplification fragments for D-genome markers in tetraploid species was established (see Supplementary Ma- terial 1).

Genotyping of introgression lines of wheat and initial parental forms indicates that all the studied lines had recombination events involving chromosomes of wheat relatives or their fragments (see Table 1). At the same time, the frequency of substitutions and translocations and the length of introgressed fragments depend on the hybrid combination and the direction of crossing (see Table 1, Supplementary Material 2). Comparison of the amplification spectra of SNP and SSR markers demonstrated the presence of the wild emmer genetic material in most common wheat chromosomes, except for line 29 (Rassvet × T. dicoccoides k-5199). Only five fragments were found in the genome of this line, and their localization coincides with the data of cytological analysis. Perhaps this is due to the direction of crossing, since in this case the wheat variety was used as a maternal component in contrast to the lines of the T. dicoccoides × Festivalnaya combination, where bread wheat was used as a pollinator.

Two lines from the T. dicoccoides × Festivalnaya combination (11-1 and 13-3) stabilized at the tetraploid level of ploidy; however, the nature of the recombination events of these lines was different. Fragments of introgression in line 13-3 are, as a rule, longer than in line 11-1. In line 11-1, according to molecular analysis, a complete replacement of the long arms of chromosomes 1B, 4A, 5B, 6A, and 7A is assumed; in other cases, inserts of foreign chromatin were insignificant (see Supplementary Material 2). In lines 15-7-2 and 16-5, alien chromatin was not found in chromosome 6A and 5A, respectively, and both lines have no introgressions in chromosome 2A.

However, these lines differed in the localization and extent of introgression fragments. Thus, in line 16-5, the inclusion of the genetic material of the T. dicoccoides B-genome was carried out in the form of larger fragments than in line 15-7-2 (see Supplementary Material 2). As for recombination events in the genome A, in line 16-5 a significant part of the 7А chromosome is replaced by the T. dicoccoides chromosome; 3AS and 4AL also originated from wild emmer. In the remaining A-genome chromosomes, T. dicoccoides segments were of small length and were found mainly in long arms. In line 15-7-2, small introgression fragments in the A-genome were found only in the long arms of chromosomes. An exception was chromosome 3A in which foreign chromatin was found in both arms (see Supplementary Material 2).

Line 206-2-2 (Pitic S62 × T. dicoccum k-45926) contained the genetic material of domesticated emmer in all chromosomes of A- and B-genomes; in line 213-1-2, introgression was not detected in chromosomes 1A, 4B, and 6B. Recombination events in the lines of this cross combination were of similar character (see Supplementary Material 2). For lines 1-3 and 2-7 of T. dicoccum k-45926 × Festivalnaya, molecular analysis did not show the presence of the alien genetic material only in chromosome 4B, while in chromosomes 1A, 2A, 2B, 3B, 4A and 7B fragments of domesticated emmer were localized in different arms (see Supplementary Material 2).

## Microsporogenesis

The analysis of the chromosome behavior at the metaphase I stage showed that the number of chromosomes forming bivalents in all introgression lines exceeded 90 % and in the majority approached 100 % (Table 2). The highest level of chromosome pairing was noted in line 16-5 – 100 %. Only one cell with two univalents out of all studied pollen mother cells (PMC) was found in lines 29 and 1-3; single cells with two univalents were found in lines 13-3, 15-7-2, 213-1-2, and 2-7. In line 11-1, 73.3 % of cells with disorders were found out of the number of analyzed PMCs, and the number of univalents in them varied from two (33.3 %) to six (10.0 %). It should be noted that line 11-1 is also characterized by the highest number of open bivalents among the studied genotypes, which indicates weakening of chromosome pairing (see Table 2). The subsequent stages of meiosis proceeded with minor disturbances, which led to the formation of normal tetrads at the final stage (Table 3).

**Table 2.**
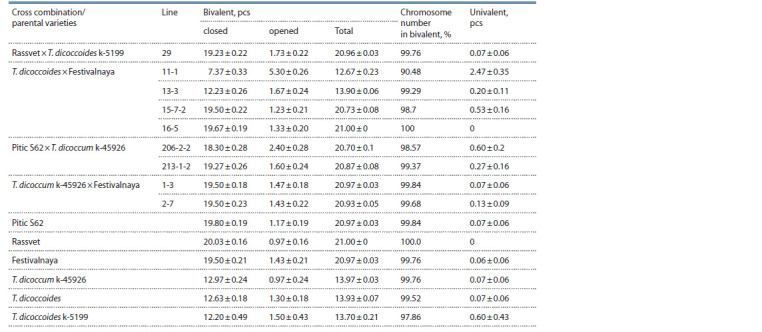
Average frequencies of different chromosome associations in metaphase I of F10 common wheat introgression lines and their parents

**Table 3.**
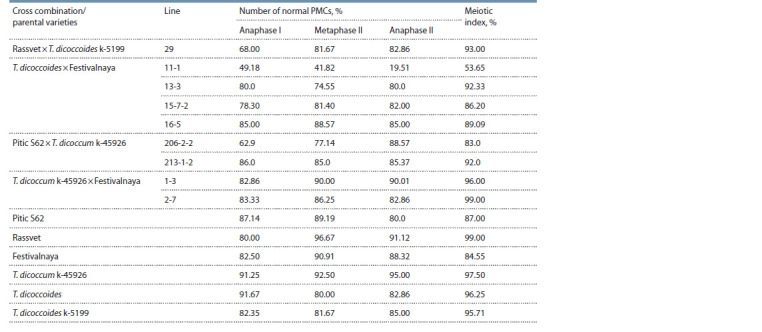
Characterization of meiotic stages in common wheat introgression lines and their parents

The exception was line 11-1 with the meiotic index of only 53.65 %, which is consistent with the data obtained from the analysis of the behavior of chromosomes at the metaphase I. It is the line that is characterized by the lowest level of synapsis among the studied introgression lines (see Table 2). Lines with a high rate of chromosomal associations in the early stages of meiosis, as a rule, had a higher value of the meiotic index (83.0–99.0 %).

At the final stage of meiosis, along with normal tetrads, abnormal tetrads with micronuclei of various sizes are formed, the number of which varied in the studied material from 1 to 6, but most often tetrads with 1–2 micronuclei were formed. It should be noted that the spectrum of abnormalities in more stable lines was much smaller. For example, in line 2–7 with a high meiotic index, only one cell with one micronucleus was found. PMCs containing six micronuclei were noted only for the least stable lines 11-1 and 206-2-2, and their frequency was very low (0.91–2.0 % of the total number of analyzed cells). In line 13-3, single triads and pentads were observed. The number of cells with such disorders was only 1.11 %. The appearance of triads is often explained by the presence of an autonomous spindle at metaphase I or metaphase II, the absence of kinetochore fibrils, or abnormal premature cytokinesis in prophase II (Sosnikhina et al., 2007).

It should be noted that a sufficiently high level of chromosome synapsis was already established by us in F2 hybrids from crossing emmer wheat with common wheat varieties (Orlovskaya et al., 2010). The number of chromosomes included in the bivalents in these hybrids was at the level of 90.9–99.3 %. As a rule, only two chromosomes did not enter the mating process. Despite the rather high level of chromosome pairing of F2 hybrids in metaphase I, the subsequent stages of meiosis in this generation proceeded with significant abnormalities. As a result, the percentage of normal tetrads (meiotic index as an indicator of the normal meiosis) was very low, ranging from 8 to 20 %. The obtained results indicate that by the tenth generation there was a significant stabilization of the meiosis, which ensures the formation of a sufficient number of functional gametes for the successful reproduction of the developed hybrid material.

## Discussion

The data obtained indicate a high frequency of introgression of the genetic material of emmer wheat into the genome of the common wheat cultivar. It should be noted that the data of SNP and SSR analyses confirm the results of differential staining of chromosomes. Thus, the highest frequency of incorporation of the genetic material of T. dicoccum and T. dicoccoides into the common wheat genome was found for chromosomes 1B, 2B, and 3B, both according to the results of molecular analysis and C-banding.

Identification of introgressions in chromosomes of the A-genome using C banding, as already noted, is difficult due to the small number of diagnostic blocks and low polymorphism of A-genome chromosomes. In addition, wild and domesticated emmer wheat are tetraploid (2n = 4x = 28) with the BBAA genomic structure, where both genomes are homologous to the corresponding T. aestivum genomes (2n = 6x = 42; BBAADD genome). As a result, these species have the same pattern of genome-specific C-bands, and the differences necessary for monitoring of alien introgressions may only concern permanent blocks of heterochromatin that are polymorphic in size, as well as non-permanent C-bands that vary in the presence/absence and size. This greatly complicates the detection of the introgression of emmer chromatin in the common wheat genome by C-banding. Therefore, for a more accurate description of chromosomal rearrangements, it is optimal to use both molecular and cytological methods. This has been demonstrated in different studies where hybrid forms from crossing different types of wheat, barley and triticale were investigated (Silkova et al., 2006; Mattera et al., 2015; Adonina et al., 2022).

The results of marker analysis indicate that the level of polymorphism and information content of SNP markers is lower compared to SSR markers (see Supplementary Material 1). This is supported by literature data obtained on various plant species (Singh et al., 2013; Garcia et al., 2018; Tereba, Konecka, 2021). However, a decrease in information content is compensated by a higher level of coverage of molecular genetic maps of chromosomes with SNP markers and a low level of null alleles in distant species.

A high level of SSR marker polymorphism is noted in most studies on the genetic diversity of varieties and hybrid forms of cereal crops (Jlassi et al., 2021; Pour-Aboughadareh et al., 2022). According to many authors, the amplification of the D-genome specific SSR markers in the genomes of alien species is 10–30 % (Salina et al., 2006; El-Rawy, Hassan, 2021).

It can be noted that recombination events in lines developed with the involvement of wild emmer occurred much more often in the long arms of chromosomes, which is consistent with the data obtained in the study of SNP polymorphism of 445 recombinant lines from crossing durum wheat with a sample of wild emmer (Jorgensen et al., 2017).

The high frequency of introgressions of the genetic material of T. dicoccum and T. dicoccoides into the genome of T. aestivum revealed in this work is a consequence of the similarity of the subgenomes of common wheat and emmer. Studies by a large group of scientists on the comparative analysis of the A-, B-, D-genomes of common wheat and its diploid and tetraploid relatives showed a high degree of homology between the corresponding genomes of related species (Petersen et al., 2006; IWGSC, 2014). At the same time, obvious differences between the species of the genus Triticum have been identified in the form of the loss of genetic material, the formation of new genes, and duplications resulting from evolutionary processes (IWGSC, 2014; Bariah et al., 2020). In addition, some studies have found silencing or alteration of gene function (Ozkan et al., 2001; Kashkush et al., 2002; Feldman, Levy, 2012). During the cultivation of wild emmer, changes were found both in morphological features and in the structure of the genome. For example, the genome size of domesticated emmer was slightly reduced compared to wild emmer (12.87 and 12.91 pg, respectively) (Eilam et al., 2008). All this leads to differences in the nucleotide sequence in the homologous chromosomes of T. aestivum and T. dicoccoides, which in turn affects the frequency of recombination events.

In our study, the lowest level of introgression was noted for chromosome 2A; for two out of five lines, no rearrangements were found in chromosomes 3A (see Table 1), which is consistent with the literature data. Thus, the analysis of the nucleotide polymorphism of the A- and B-genomes of T. aestivum and T. dicoccoides revealed significant differences between the 2A chromosomes of these related species. The average nucleotide polymorphism in this chromosome of T. aestivum and T. dicoccoides was 0.56 and 0.83, respectively, and the average number of haplotypes per locus was 1.85 and 2.25, respectively (Akhunov et al., 2010). Significant differences were also found between chromosomes 3A and 4A of T. aestivum and T. dicoccoides compared to other chromosomes (Akhunov et al., 2010). It should be noted that a comparative analysis of the subgenomes of common wheat and diploid and tetraploid relatives of T. aestivum showed some differences in the sequence of genes on chromosomes 2A and 7B, which suggests the presence of small translocations or introgressions that occurred during evolution (IWGSC, 2014).

Taking into account the homoeology of the A- and B-genomes of T. aestivum with similar genomes of T. dicoccoides and T. dicoccum, one should expect a rather high level of chromosome pairing in metaphase I of meiosis of F1 hybrids followed by the formation of reciprocal exchanges between the regions of homologous wheat and emmer chromosomes. Analysis of the metaphase I stage revealed a high level of bivalent pairing of chromosomes in all studied F10 introgression lines (see Table 2).

It is known that the long arm of chromosome 5B contains the Ph1 locus, which is the main regulator of chromosome synapsis and prevents mating of homoeologs (Riley, Chapman, 1958; Naranjo, 2012). The lack of activity of this locus in diploid relatives of wheat indicates its occurrence as a result of structural changes in chromosome 5B after polyploidization (Chapman, Riley, 1970). Ph1 candidate gene (C-Ph1) has been identified, whose silencing resulted in formation of multivalents (Bhullar et al., 2014). Three homoeologous copies of C-Ph1 were found on chromosomes 5A, 5B and 5D.

The nucleotide sequence of homoeologous genes has a similarity of about 90 % and differs, as a rule, by insertions and deletions, which lead to changes in the amino acid sequence of the protein. In addition, significant differences were found in the level of expression of homoeologous genes during different stages of the meiotic cycle. For C-Ph1 on chromosome 5B, the highest level of activity was noted during metaphase I, the lowest level was observed during anaphase I and the absence of activity at subsequent stages. The 5A copy was expressed during anaphase I, dyad and tetrad stages. The 5D copy showed the highest activity during early stages of meiosis (interphase and prophase stages) (Bhullar et al., 2014). In our study, all lines, with the exception of line 13-3, revealed structural changes in the long arm of chromosome 5B; however, as a rule, these transformations do not cause significant weakening of homologue synapsis. A negative effect of chromatin introgression of the wild emmer 5B chromosome is most pronounced in line 11-1, which may be due to the length of the translocated fragment (Supplementary Material 2).

For lines developed with the involvement of domesticated emmer, a higher meiotic index was noted than for lines based on wild emmer. The maximum meiotic index was found for lines 2-7 and 1-3 of T. dicoccum k-45926 × Festivalnaya (see Table 3). This is probably due to the closer similarity between the A and B genomes of T. aestivum and T. dicoccum. The highest stability among the lines with the insertion of the wild emmer genetic material was found for line 29 (93.0 %), the level of chromosome pairing of which was one of the highest (see Table 2). Line 16-5 with 100 % of the number of chromosomes included in bivalents was characterized by a lower value for this indicator (89.09 %). This fact can be explained by differences in the number of introgressed fragments in lines 29 and 16-5. Thus, line 29 contains fragments of the foreign genome in 5 chromosomes, and line 16-5 in 12 chromosomes

Correlations between the number of alien fragments and the meiosis abnormality degree are also shown in the publications of other researchers (Gordeeva et al., 2009; Zeng et al., 2013). In addition, one should not exclude an influence of the genotype of the original wheat variety on the cytological stability of the studied lines. Thus, the meiotic index of the Rassvet variety (99.0 %) was higher than that of the Festivalnaya variety (84.55 %), and line 29 developed with the involvement of a more stable variety had the highest percentage of normal tetrads among introgression lines (see Table 3).

All lines with a high meiotic index contain alien fragments in chromosomes 1B, 2B, and 3B; most lines – in chromosomes 5B and 6B and are of interest for expanding of the wheat gene pool. For example, a functional allele of the Gpc-B1 gene associated with a high content of protein and some microelements in wheat grain were found on chromosome 6B of wild emmer (Uauy et al., 2006). In wild emmer accessions, genes associated with an increased content of total grain protein were also found on chromosomes 2A, 5B, 6B, and 7B (Ohm et al., 2010). Powdery mildew resistance genes were mapped on chromosomes 2B (Zhang et al., 2010) and 5B (Xue et al., 2012) of wild emmer, and drought resistance genes were mapped to 5B loci (Akpinar et al., 2015). Currently, using the GWAS and RNA-seq approach, genetic factors associated with the accumulation of protein and minerals in wild emmer have been identified (Liu et al., 2021; Gong et al., 2022).

## Conclusion

Thus, the results of molecular cytogenetic analysis of introgression wheat lines indicate that the insertion of the genetic material of wild and domesticated emmer, whose genomes are homologous to common wheat genomes, occurs mainly in the form of whole arms or large fragments in the chromosomes of the B-genome and less extended inserts in genome A. At the same time, the highest frequency of introgression emmer genome was observed in chromosomes 1A, 1B, 2B, and 3B.

The use of karyotyping methods in combination with screening of hybrid lines with molecular markers makes it possible to obtain extensive information on chromosomal rearrangements and the sizes of alien introgressed fragments. Analysis of the final stage of meiosis showed a high level of cytological stability in the vast majority of studied wheat lines. It should be noted that the lines characterized by an insignificant number of anomalies at the early stages of microsporogenesis, as a rule, had a higher value of the meiotic index. Introgression lines with a normal course of meiosis will be used in further studies (identification of genes that control resistance to biotic and abiotic stressors, high grain quality, etc.) in order to identify promising material for inclusion in the breeding process.

## Conflict of interest

The authors declare no conflict of interest.
